# The Second Edition of Text-Book Hygiene

**Published:** 1891-03

**Authors:** 


THE SECOND EDITION OF TEXT-BOOK HYGIENE. A comprehensive treatise on the Principles of Preventive Medicine from an American Standpoint. By George H. Rohe, M. D., Professor of Obstetrics and Hygiene in the College of Physicians and Surgeons, Baltimore; Director of the Maryland Matemite; Member of the American Public Health Association; Foreign Associate of the Societe Francaise D'Hygiene, of the Society des Chevaliers Sauteurs des Alpes. Published by F. A. Davis, Philadelphia.

Dr. Rohe in his preface claims "nothing new, and hopes that nothing may be found untrue." This at least, is modest, and although the reviewer can vouch for the first, and assert the second, yet, there remains much more to be said in favor of the volume; we are somewhat oriental in our appreciation of reading matter, that there is "value in all printing," and say, that though avowedly a compilation, yet the compiler deserves praise in the effort to set forth so lucidly, the salient principles of hygiene, in all its different branches.

Reasoning from observation and experience, that work is most valuable to the public which will give the reader the most information in the least time, and with a minimum exertion.

This Dr. Rohe has succeeded in doing, and therefore, from an American standpoint, the volume recommends itself. The author has certainly digested the science of Hygiene, and given the same to the public in an easily assimilable form.

